# Supplementary material for: Sendai Virus Infection Induces Expression of Novel RNAs in Human Cells
Source: Sci Rep. 2018 Nov 14;8:16815. doi: 10.1038/s41598-018-35231-8 (PMC6235974; doi:10.1038/s41598-018-35231-8)
Supplement: Supplementary file 1 — Supplementary Figures [file 41598_2018_35231_MOESM1_ESM.docx]

Sendai Virus Infection Induces Expression of Novel RNAs in Human Cells

Roli Mandhana, Curt M. Horvath^*^

*Department of Molecular Biosciences*

*Northwestern University, Evanston, IL 60208, USA*

^*^Corresponding author

Tel: (847) 491-5530

Fax: (847) 491-0848

Email: [*horvath@northwestern.edu*](mailto:horvath@northwestern.edu)


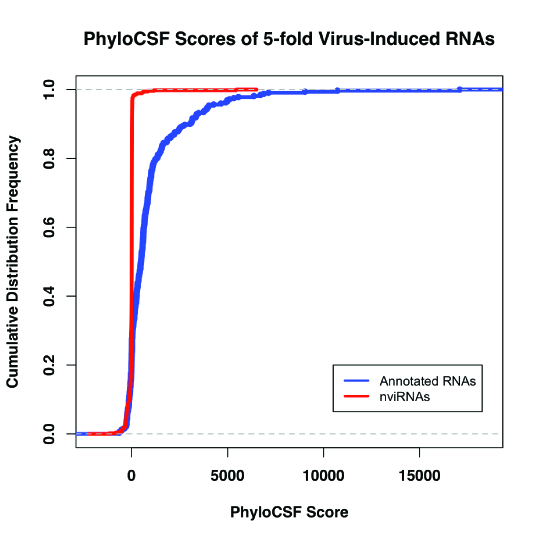


Supplementary Figure S1 (related to Figure 4a) - The cumulative distribution frequency of PhyloCSF scores for the 5-fold induced previously-annotated RNAs (blue) and nviRNAs (red).


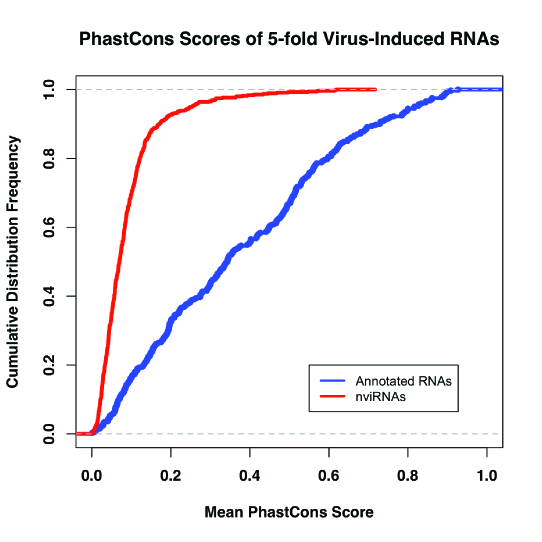


Supplementary Figure S2 (related to Figure 4d) - The cumulative distribution frequency of the mean PhastCons score for the 5-fold induced previously-annotated RNAs (blue) and nviRNAs (red).
